# The 86th Annual Meeting of the Japanese Orthopaedic Association

**DOI:** 10.1007/s00776-013-0375-4

**Published:** 2013-03-13

**Authors:** Mitsuo Ochi

**Affiliations:** Department of Orthopaedic Surgery, Integrated Health Sciences, Institute of Biomedical & Health Sciences, Hiroshima University, Hiroshima, Japan

It is a tremendous honor to host the 86th Annual Meeting of the Japanese Orthopaedic Association (86th JOA) in Hiroshima. The meeting will take place from May 23 to 26, 2013, in four venues that are all within 10 minutes’ walk of the city center: Hiroshima Green Arena, the Rihga Royal Hotel Hiroshima, NTT Cred Hall, and Mielparque Hiroshima. Hiroshima is a thriving, vibrant city, having risen from the destruction of the atomic bomb in WWII, and today serves as a city that is dedicated to permanent world peace and the elimination of nuclear weapons. As Japan looks to the future and aims to revitalize itself in the wake of the Great East Japan Earthquake, the opportunity to host the 86th JOA carries a profound significance and sense of mission for us.

The earthquake and tsunami of March 11, 2011 took many precious lives and triggered the accident at the Fukushima Daiichi nuclear power plant. How well did we apply experience gained in the Kobe earthquake of January 17, 1995 and other past disasters to this event? While such lessons were put to good use in many aspects of the response, I understand that this was not always the case. The process of disaster recovery has only just begun, but I believe we need to verify past facts and remember new and painful experiences so that we can make use of them in the future. Over the course of history, humans have had the opportunity to learn a great many things. Have we made sufficient use of such lessons? The theme for the 86th JOA is ‘Let’s learn from history,’ by which we mean shedding light on the past and understanding the present to seek new solutions for the future. In orthopaedics, too, we hope to clarify changes from past practice and learn from the lessons of the past to address the pressing issue of anticipating future treatments for locomotive organ disorders.

During the 20th century, Japan made remarkable contributions to medical care. The long list of achievements includes developments in minimally invasive surgery using arthroscopy, tissue transplant via microsurgery, and advances in cervical laminoplasty. Endoscopes for examining the body cavity via the mouth, anus or urethra were developed long ago, but Professor Kenji Takagi and his team at the University of Tokyo were the first to devise a method for inserting an endoscope into a closed cavity via a scalpel incision [1]. They began researching arthroscopy in 1918, and by 1931 had successfully conducted arthroscopy of the knee. Dr. Masaki Watanabe of Tokyo Teishin Hospital improved the technique, introducing the Watanabe no. 21 arthroscope in 1959 and conducting the first meniscectomy under arthroscopy in 1962 [2]. In 1968 Dr. Shigeo Komatsu and Professor Susumu Tamai of Nara Medical University carried out the first successful replantation of a completely cut-off thumb using microvascular anastomoses [3]. Professor Kiyonori Harii and his team achieved the first clinical success in free gracilis muscle transplantation with microneurovascular anastomoses for the treatment of facial paralysis in 1975 [4]. Professor Yoshikazu Ikuta and his colleagues at Hiroshima University reported the first free muscle transplantation by microsurgical technique to treat severe Volkmann’s contracture in 1976 [5]. Dr. Yoshito Kirita of Kyoto University devised a technique for using a high-speed drill to conduct extensive simultaneous multisegment laminectomy, a procedure for removing a portion of vertebral bone, in 1968 [6]. Three years later Professor Susumu Hattori of Yamaguchi University developed z-shaped laminoplasty, which preserves the posterior elements [7]. In 1977, Professor Kiyoshi Hirabayashi of Keio University further developed these methods to devise expansive open door laminoplasty, a procedure that became widely used [8]. Professor Kenya Tsuge of Hiroshima University published a paper in 1975 detailing a new technique for intra-tendinous tendon suture to repair damaged flexor tendons in the hand [9]. Described in many medical textbooks, the Tsuge stitch uses looped sutures to preserve intra-tendinous blood flow and minimize tendon trauma. In 1956 Professor Atsuto Nishio of Kyushu University developed transposition osteotomy of the acetabulum to treat acetabular dysplasia [10], and in 1968 Professor Hiroshi Tagawa of Tokyo Women’s Medical University carried out the first rotational acetabular osteotomy (RAO) [11]. Professor Yoichi Sugioka of Kyushu University achieved another first in this field in 1972, when he conducted a transtrochanteric rotational osteotomy of the femoral head (TRO) [12]. The brilliant techniques devised by these pioneers are still being used and refined in arthroscopic joint surgery, tissue reconstruction to repair deficits due to trauma and tumor excision, microscopic spine surgery, and joint preservation surgery using osteotomy.

Needless to say, one of the highlights of the 21st century for the Japanese Orthopaedic Association has been the honor of seeing JOA member, Professor Shinya Yamanaka awarded the Nobel Prize in Physiology or Medicine, an accomplishment that will be talked about for many years to come. However, a gradually growing number of young people are unaware of the many tremendous achievements for our forebears in Japan. We need to build the foundations of future advances by telling the younger generation about such pioneers’ sense of mission and the paths and methodologies they used to attain valuable research results through persistent creativity and ingenuity.

The slowdown in the Japanese economy and strikingly rapid growth in other East Asian economies seems to be reflected in gradually diminishing prominence for Japan in international journals. Now is the time to review Japan’s history, gain a sound understanding of the current situation, look to Asia and the wider world, and become more globally active. In my Congress President’s address, I will share some personal thoughts on orthopaedic developments and issues currently facing the field.

The keynote lecture on May 23, the first day of the meeting, is entitled ‘Let’s learn from history—from the past to the future of orthopaedics’, and will be delivered by two speakers who have been driving forces within JOA: former JOA president, Professor Takahiro Ochi and Professor Yoshiaki Toyama of the Department of Orthopaedic Surgery, Graduate School of Medicine, Keio University. The program for day 3 (May 25) features ten lectures with two speakers apiece addressing the theme of ‘Let’s learn from history’, linking the past and future of fields including knee, hip, spine, hand, and shoulder treatments as well as musculoskeletal tumors.

Five guest speakers will deliver special lectures. Incidence trends in orthopaedic disorders alter dramatically as times change, and with Japan now facing a ‘super-aged’ society, locomotive syndrome has been identified as a national affliction in urgent demand of appropriate responses. Former JOA president, Professor Kozo Nakamura will speak about this syndrome. The locomotive organs are closely connected with regenerative medicine, and Professor Teruo Okano, Director of the Institute of Advanced Biomedical Engineering and Science at Tokyo Women’s Medical University, will share his dreams in a lecture entitled ‘Clinical applications of cell sheet regenerative medicine’. Toshiharu Furukawa, Professor in the Law School and also School of Medicine at Keio University and a member of the House of Councillors, will present a vision for Japanese medicine in his lecture on ‘Trend of healthcare policy in orthopaedics’. Two lectures will focus on the growing role of women in orthopaedics: ‘Always think positive’, presented by Fumiko Yonezawa, Professor Emeritus at Keio University, and ‘Raising woman leaders of the next generation’, delivered by Michiko Go, former president of Ochanomizu University.

The speaker for our cultural lecture will be Tatsuru Uchida, award-winning author of works including *Shikaban: Yudaya Bunkaron* (Private Edition: Study of Jewish Culture) and the best-selling *Nihon Henkyoron* (Japan as a Peripheral Country). In rotation with Tokyo University Professor Emeritus Takeshi Yoro, Professor Emeritus Uchida writes the opening column in the weekly magazine *Aera* covering topics including medicine, education, justice, and religion, and will share his own unique insights.

Based on proposals made by JOA committees, related associations, and the host university, the symposium and panel discussion program will include two special symposia, 36 symposia, and 18 panel discussions. All panelists have been appointed. The special symposia are entitled ‘Learning from mega disasters’ and ‘How Orthopaedics in Japan looks, based on clinical experience abroad’. Key topics covered by other symposia include ‘Latest research on locomotive organs regeneration’, ‘Front lines of ACL reconstruction’, ‘Femoroacetabular impingement (including secondary FAI after osteotomy)’, ‘Minimally invasive surgery for lumbar spinal disorders’, ‘Minimally invasive surgery for bone and soft tissue sarcomas’, ‘Pathogenesis and treatment of lateral epicondylitis of the elbow’, ‘Pathogenesis and surgical treatment of rotator cuff tears’, ‘Etiology and current conservative therapies for osteoarthritic knee’, ‘Updated knowledge of idiopathic osteonecrosis of the femoral head’, ‘Advances in diagnostic imaging methods of the spine’, ‘Ultrasonographic assessment in orthopaedics‘, ‘Future strategies with new therapeutic drugs for bone and soft tissue tumors’, and ‘Medication for chronic pain in the locomotive organs: indications and limitations’.

The Specialty Day, an initiative first introduced at the 82nd JOA, will take place on the second day of the meeting (May 24). The program for this day will incorporate the latest information on specific areas including the spine, knees, hips, shoulders, hands, elbows and feet as well as fields such as fractures, paediatrics, bone and soft tissue tumors, rheumatism, osteoporosis, and pain. Video presentations will illustrate the realities and knacks of surgical technique in these fields.

Twenty-eight presentations feature overseas lecturers, with two speakers per session. In some cases both lecturers are from abroad, while in others an overseas guest is teamed with a Japanese speaker. Focusing on international topics in a broad range of fields, themes for these sessions include regenerative medicine, the bone and joint decade, and tips for writing papers in English.

Forty educational and training lectures will cover a wide array of topics including the future of the orthopaedic specialist system, changes in treatment methods over the course of orthopaedic history, the latest research, medical policy, and medical disputes. On the fourth day of the meeting (May 26) Hiroshima University Professor Emeritus Kenya Tsuge, Osaka University Professor Emeritus Keiro Ono, Kyoto University Professor Emeritus Takao Yamamuro, Juntendo University Professor Emeritus Yasuo Yamauchi, and Kochi University Professor Emeritus Hiroshi Yamamoto will deliver their messages to young orthopaedic surgeons.

A full program of 61 sponsored seminars comprises 5 morning, 40 luncheon and 16 evening seminars. Luncheon seminars feature a set theme on each day of the meeting: presentations by overseas guests on day one, ‘Japan’s historical role: contributions to the progress of orthopaedics’ on day two, ‘Future perspective’ on day three, and ‘Clinical case review’ on day four.

A record total of 2,153 free papers were submitted, 2,022 in Japanese and 131 in English. Our sincere thanks go to all JOA members who submitted papers. In order to maintain an acceptance rate (65 %) in line with other years we have made every possible effort to expand the number of presentations, with posters being changed after the second day. The best poster presentations will be eligible for prizes including the Hiroshima Prefecture Governor’s Award, the Hiroshima Mayor’s Award, and the Outstanding Poster Award.

As in other years, a short course for training leaders will be held on the afternoon of the final day. The instructors will be JOA Vice President Naoyuki Ochiai and Kazue Nakajima, Director and Clinical Professor, Department of Patient Safety and Quality Management, Osaka University Hospital.

We will do our utmost to ensure that the 86th JOA provides opportunities to learn from many aspects of history, sheds light on the development of orthopaedics in Japan, and presents a vision for the future of orthopaedics in this country. Hiroshima offers an archetypal Japanese landscape, where mountains, valleys, basins, plains, rivers and ocean weave a complex topography. Blessed with a rich natural environment stretching from the islands of the Seto Inland Sea to the Chugoku Mountains in the hinterland of the city, Hiroshima is also home to two World Cultural Heritage sites: the Hiroshima Peace Memorial (Genbaku Dome) and Itsukushima Shrine, renowned for its vermillion Torii gate and buildings that appear to be floating on the sea and its Heike Nokyo scrolls. Close to the meeting venues is a replica of Hiroshima Castle, originally constructed by Terumoto Mori in 1589. Also nearby is Shukkeien Garden, construction of which began in 1620 at the orders of Nagaakira Asano, first feudal lord of the Hiroshima area. The garden was built by his principal retainer, Soko Ueda, a famous master of the tea ceremony. On the north side of the Rihga Royal Hotel Hiroshima is the Hiroshima Museum of Art, with a collection of modern European art focusing on French impressionists. We hope that meeting participants will take the chance to visit these sites during their free time.

Yu Fujikawa, who completed an important history of Japanese medicine in 1904, was born in Hiroshima at the end of the Edo Period and studied at the Hiroshima Prefectural Medical School. Together with other scholars including Shuzo Kure, who was born into the house of a doctor serving the Hiroshima feudal domain, and Shiro Amako, a friend of the famous novelist Soseki Natsume, he founded the Geibi Medical Society (now the Hiroshima Medical Association), which greatly contributed to improving medical standards in the Hiroshima region during the Meiji Period. Fujikawa was a close friend of Ogai Mori, and helped in the writing of some of Ogai’s major works, including the biographical novel *Izawa Ranken*. As well as being physician to the feudal lord of Bingo Fukuyama district, Izawa was a Confucian scholar and composer of poetry in the Chinese style, and a close associate of Sanyo Rai, the author of *Nihon Gaishi* (Unofficial history of Japan), Chazan Kan, a leading Confucianist and poet of the Edo era, and Nanpo Ota, who wrote under pen names including Shokusanjin. The Rai Sanyo Shiseki Museum, located near the meeting venues, recreates the former residence of Sanyo Rai and exhibits examples of calligraphy that evoke the lively interaction and purposefulness typical of the cultured circles of Geibi District in days gone by. I hope you will take the opportunity to suspend the passage of time for a fleeting moment to sense the lingering presence of the pioneers of Japanese medical history and to look back over our past while also letting your thoughts run ahead to the future.

The entire Hiroshima region is ready with a warm welcome for participants in the 86th JOA. We look forward to seeing you all in May 2013.

## References

[1] Takagi K. Practical experience using Takagi’s arthroscope. Nippon Seikeigekakai Zasshi (J Jpn Orthop Assoc). 1933;8:132 (in Japanese).

[2] Watanabe M, Takeda S. The number 21 arthroscope. Nippon Seikeigekakai Zasshi (J Jpn Orthop Assoc). 1960;34:1041 (in Japanese).

[3] Komatsu S, Tamai S (1968). Successful replantation of completely cut-off thumb, case report. Plast Reconst Surg.

[4] Harii K, Ohmori K, Torii S (1976). Free gracilis muscle transplantation, with microneurovascular anastomoses for the treatment of facial paralysis. A preliminary report. Plast Reconstr Surg.

[5] Ikuta Y, Kubo T, Tsuge K (1976). Free muscle transplantation by microsurgical technique to treat severe Volkmann’s contracture. Plast Reconstr Surg.

[6] Kirita Y. Posterior decompression for cervical spondylosis and OPLL. Shujutsu, 1976; 30:287–302 (in Japanese).

[7] Oyama M, Hattori S, Moriwaki N. A new method of posterior decompression. Chubuseisaisi 1973;16:792 (in Japanese).

[8] Hirabayashi K, Watanabe K, Wakano K (1983). Expansive open-door laminoplasty for cervical spinal stenotic myelopathy. Spine.

[9] Tsuge K, Ikuta Y, Matsuishi Y (1975). Intra-tendinous tendon suture in the hand — a new technique. Hand.

[10] Nishio A. Transposition osteotomy of the acetabulum for the treatment of congenital dislocation of the hip. Nippon Seikeigekakai Zasshi (J Jpn Orthop Assoc). 1956;30:482–4 (in Japanese).

[11] Ninomiya S, Tagawa H (1984). Rotational acetabular osteotomy for the dysplastic hip. J Bone Joint Surg Am.

[12] Sugioka Y (1978). Transtrochanteric anterior rotational osteotomy of the femoral head in the treatment of osteonecrosis affecting the hip: a new osteotomy operation. Clin Orthop Relat Res.

